# PET Imaging of FSHR Expression in Tumors with ^68^Ga-Labeled FSH1 Peptide

**DOI:** 10.1155/2017/2674502

**Published:** 2017-08-23

**Authors:** Donghui Pan, Guifeng Liu, Yuping Xu, Yanting Wang, Yuanyuan Yue, Lizhen Wang, Junjie Yan, Xinyu Wang, Runlin Yang, Min Yang

**Affiliations:** ^1^Key Laboratory of Nuclear Medicine, Ministry of Health, Jiangsu Key Laboratory of Molecular Nuclear Medicine, Jiangsu Institute of Nuclear Medicine, Wuxi, Jiangsu 214063, China; ^2^Department of Radiology, China-Japan Union Hospital of Jilin University, Changchun, Jilin 130033, China; ^3^Nanjing Medical University, Nanjing, Jiangsu 210029, China; ^4^Zhengzhou University, Zhengzhou, Henan 450001, China

## Abstract

FSHR is an appealing target for cancer theranostics. Radiolabeled FSH1 and its derivatives have shown potential to in vivo detect FSHR expression. However, moderate labeling yields (~50% nondecay-corrected) may partially limit their wide use. ^68^Ga is an excellent PET nuclide due to availability, nearly quantitative reaction, and short physical half-life. In this study, ^68^Ga labeled FSH1 peptide was developed for imaging of FSHR in cancers. In vitro studies and MicroPET imaging were performed in PC-3 prostate tumor model. [^68^Ga] Ga-NOTA-MAL-FSH1 can be produced within 20 min with 93.2 ± 2.1% yield and the radiochemical purity was greater than 95%. It showed that [^68^Ga] Ga-NOTA-MAL-FSH1 possessed FSHR binding affinities. The tracer was stable in PBS and human serum for at least 2 hours. MicroPET imaging revealed that the PC-3 xenografts were clearly visualized and the tumor uptakes were 1.87 ± 0.10, 1.26 ± 0.06, and 0.71 ± 0.10% ID/g at 0.5, 1 h, and 2 h postinjection. The corresponding tumor to blood and tumor to muscle ratios were 1.77 ± 0.70, 7.94 ± 1.35, and 10.37 ± 1.16 and 7.42 ± 0.46, 26.13 ± 2.99, and 36.40 ± 2.54, respectively. FSHR binding specificity was also demonstrated by reduced tumor uptake of [^68^Ga] Ga-NOTA-MAL-FSH1 after coinjecting excess unlabeled FSH1 peptide. The favorable characters of [^68^Ga] Ga-NOTA-MAL-FSH1 such as convenient synthesis and specific tumor uptake warrant its further investigation for FSHR expression imaging.

## 1. Introduction

Follicle-stimulating hormone (FSH) is a central hormone in mammalian reproductive biology. It promotes the mature spermatogenesis for men and follicular maturation for women, respectively. FSH receptor (FSHR) is a glycosylated transmembrane protein belonging to the family of G-protein-coupled receptors. In adult humans and animals, low levels of FSHR were expressed only in endothelial cells of ovary and testis [1–4].

Immunostaining showed that FSHR was overexpressed in the vasculatures of various solid tumors including prostate, breast, lung, and ovarian cancer. On the contrary, healthy and nonmalignant inflammatory tissues were always FSHR negative [5]. These findings imply that the receptor may be a target for cancer detection and image guided cancer surgery. Moreover, the expression level of FSHR correlates strongly with the response of tumors to antiangiogenic therapies. One clinical study in patients found that the FSHR levels in the primary tumors correlated well with the response to antiangiogenic tyrosine kinase inhibitors (sunitinib) [6]. The percentage of FSHR stained vessels of the responsive patients (~60%) was significantly higher than those in the stable or nonresponsive group (~10% and ~7%, resp.). These characteristics render FSHR an attractive choice for tumor theranostics. Using the FSHR-targeting strategy, paclitaxel attached to FSH peptide was loaded into nanoparticles and the compound displayed antitumor effects [7]. Meanwhile, a nanographene oxide conjugated to a monoclonal antibody against FSHR was successfully developed for targeting delivery of therapeutics [8].

Besides therapy, noninvasive imaging of FSHR is of benefit to monitor biochemical changes and target abundance within a testing subject. Radiolabeled peptides toward receptors have attracted considerable interest for cancer imaging due to fast clearance, high specificity, excellent tissue penetration, and low immunogenicity [9–13]. Preliminary studies revealed that ^18^FAl labeled FSH1 and its derivatives (FSH2) possessed FSHR binding specificity and might be potential probes to in vivo detect FSHR expression [13, 14]. However, moderate labeling yields (~50% nondecay-corrected) may partially limit their wide use.

Compared with ^18^F, a metallic positron emitter, ^68^Ga, can be conveniently obtained from an in-house ^68^Ge/^68^Ga generator and independent of an onsite cyclotron. With a half-life of 68 min, it is also suitable for the pharmacokinetics of many peptides. In addition, biomolecules can be labeled with ^68^Ga by nearly quantitative reaction with macrocyclic chelators. It allows possible kit formulation and accelerated the application of the probes [15].

**Figure 1:**
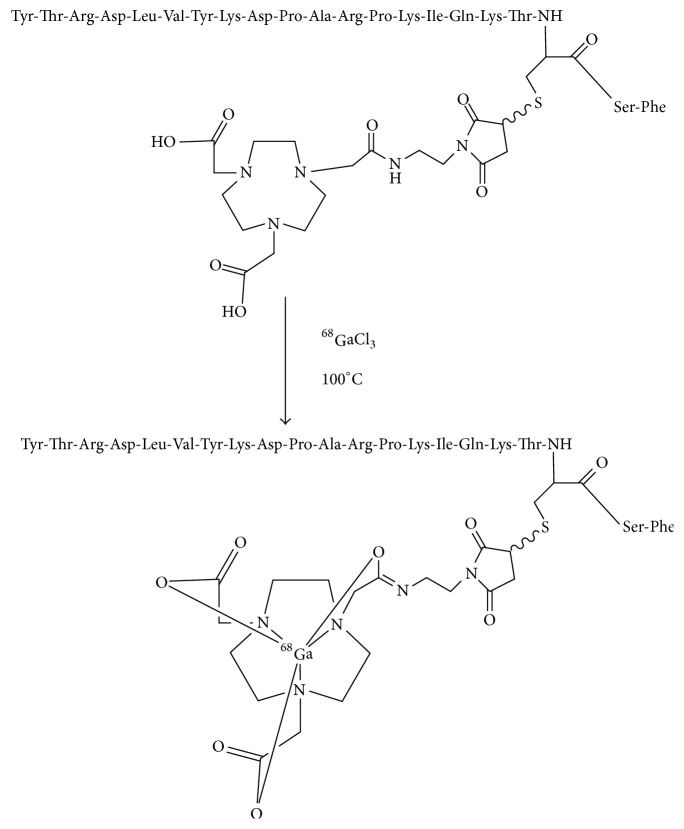
Schemes for radiosynthesis of [^68^Ga] Ga-NOTA-MAL-FSH1.

Previous studies showed that the uptake of ^18^FAl labeled FSH1 in FSHR positive PC-3 xenograft was significantly higher than those of FSH2 counterpart at 1 h postinjection (2.64 ± 0.25% ID/g versus 1.88 ± 0.02% ID/g, resp.). FSH1 peptide may be more suitable for tumor imaging than FSH2 [13, 14]. In this study, FSH1 conjugated to maleimide-NOTA, NOTA-MAL-FSH1, was labeled with ^68^Ga (Figure 1). The in vitro affinity and the in vivo tumor targeting properties of [^68^Ga] Ga-NOTA-MAL-FSH1 were also determined in PC-3 tumor models.

## 2. Materials and Methods

All commercial reagents were of analytical grade. FSH1 peptide was custom made by Apeptide Co., Ltd., (Shanghai, China). NOTA-MAL-FSH1 was synthesized according to the literature and the purity was greater than 95% [13].

The conditions for HPLC system are as follows [16]: A Waters high-performance liquid chromatography (HPLC) system with a Waters 2998 photodiode array detector (PDA) and a preparative C18 HPLC column (5 *μ*m, 250 × 10 mm, Phenomenex) was used for peptide conjugated purification. The flow is 5 ml/min, and the mobile phase changed from 95% solvent *A* (0.1% trifluoroacetic acid in water) and 5% solvent *B* (0.1% trifluoroacetic acid in acetonitrile) (0–2 min) to 35% solvent *A* and 65% solvent *B* at 35 min. The UV absorbance was monitored at 218 nm.

The purities of compounds were analyzed by RP-HPLC on a Waters Breeze system equipped with a Radiomatic 610TR flow scintillation analyzer (PerkinElmer) and a Waters 2487 dual *λ* absorbance detector. A Luna C18 column (5 *μ*m, 250 × 4.6 mm, Phenomenex) was used at a flow rate of 1 ml/min with the following buffer system: buffer *A*, 0.1% v/v trifluoroacetic acid in water; buffer *B*, 0.1% v/v trifluoroacetic acid (TFA) in acetonitrile (ACN); and a gradient of 95% buffer *A* at 0–2 min to 35% buffer *A* at 35 min.

**Figure 2:**
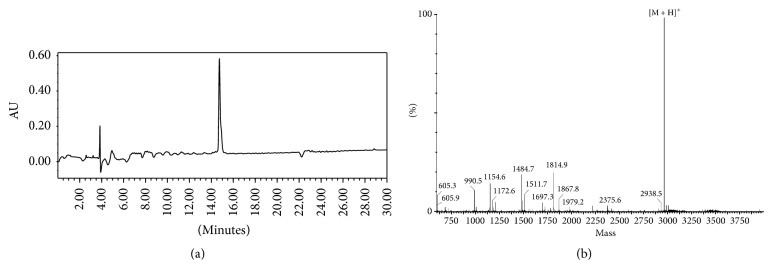
HPLC chromatogram (a) and MS spectrum (b) of NOTA-MAL-FSH1.

The HPLC chromatogram and MS spectrum of NOTA-MAL-FSH1 were listed in Figure 2. MS measured *m*/*z* 2968.5 for [M + H]^+^ (C_133_H_210_N_36_O_39_S, calculated molecular weight, 2968.1) [13]; [^68^Ga] GaCl_3_ was eluted from a ^68^Ge/^68^Ga-generator (ITG, Germany) with 0.05 M HCl. The PC-3 human prostate cancer cell line was purchased from Cell Bank of Shanghai Institutes for Biological Sciences.

### 2.1. Preparation of  ^68^Ga-NOTA-MAL-FSH1

**Figure 3:**
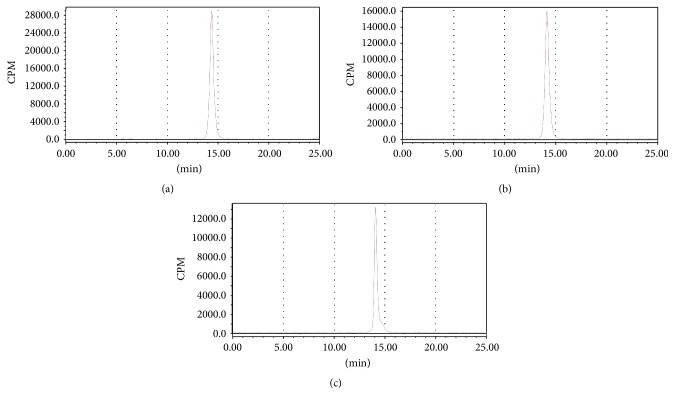
HPLC radiochromatogram for (a) purified [^68^Ga] Ga-NOTA-MAL-FSH1 and (b) [^68^Ga] Ga-NOTA-MAL-FSH1 after 2 h incubation in PBS and (c) [^68^Ga] Ga-NOTA-MAL-FSH1 after 2 h incubation in human serum.

The fresh [^68^Ga] GaCl_3_ (185 MBq) was added to 300 *μ*L 1 M HEPES buffer and followed 20 *μ*g of NOTA-MAL-FSH1 (6.7 nmol) in 10 *μ*L 0.2 M sodium acetate buffer (pH = 4). The mixture was incubated at 100°C for 10 min. After being diluted with 10 mL of deionized water, the complex was loaded into a Varian BOND ELUT C18 column. The labeled peptide was eluted with 200 *μ*L 10 mM HCl in ethanol. The product was reconstituted in 3 ml saline and passed through a 0.22 *μ*m Millipore filter into a sterile vial. Radiochemical purity was checked with the HPLC system. The retention time for ^68^Ga-NOTA-MAL-FSH1 was 14.2 min (Figure 3(a)).

### 2.2. Octanol/PBS Partition Coefficient

To an Eppendorf tube filled with 0.5 mL of the radiolabeled peptide (10 *μ*L, 370 KBq) in phosphate-buffered saline (pH 7.4), 0.5 mL of octanol was added. After stirring for 2 min at room temperature, the two layers were separated by centrifugation. Radioactivity in 100 *μ*L of each layer was measured in a *γ*-counter (Perkin-Elmer), and log *D* values were calculated [17]. Experiments were performed in triplicate.

### 2.3. Stability Studies

10 *μ*L 370 KBq [^68^Ga] Ga-NOTA-MAL-FSH1 in saline was incubated in 200 *μ*L PBS or 200 *μ*L human serum for 30, 60, and 120 min at 37°C, respectively. The stability of the labeled peptide in PBS was determined by directly injecting an aliquot of the solution into the HPLC at the preselected time points.

The serum was first mixed with 200 *μ*L acetonitrile to precipitate the proteins. Subsequently, after centrifugation, supernatant was collected and analyzed by radio-HPLC [18]. Protein-binding fraction was calculated by dividing the radioactivity of the precipitation layer by the total activity used.

### 2.4. Cell Lines and Animal Models

PC-3 cells were cultured and grown in F-12 nutrient (Thermo Fisher) mixture supplemented with 10% (v/v) fetal bovine serum at 37°C in an atmosphere containing 5% CO_2_.

The PC-3 tumor model was generated by subcutaneous injection of 5 × 10^6^ cells into the right front flank of male athymic nude mice (4 weeks old, SLAC Laboratory Animal Co., Ltd., China).

The mice were used for MicroPET studies when the tumor volume reached 100–300 mm^3^, which took about 2 weeks. The animal experiments were conducted in compliance with the national regulations and approved by local animal welfare committee.

### 2.5. Competitive Cell-Binding Assay

To obtain a homogenous mixture, equivalent of ^nat^GaCl_3_ was added to [^68^Ga] Ga-NOTA-MAL-FSH1 and the final solution incubated again for 10 min. Binding affinities toward the FSHR for [^68^Ga] Ga-NOTA-MAL-FSH1 were determined in a competitive binding assay on PC-3 tumor cells using FSH1 as a receptor ligand [19]. PC-3 cells in 6-well plates (2 × 10^5^ cells per well) were cultured until confluency. On the day of assay, cells were washed with binding buffer (RPMI, 0.5% bovine serum albumin). A range of 1 to 5000 nM FSH1 in binding buffer and 370 KBq [^68/nat^Ga] Ga-NOTA-MAL-FSH1 were added to each well. After incubating at 37°C for 2 h, medium was removed. Then, cells were washed with binding buffer and extracted from the wells by 0.1 M NaOH. Cell-associated radioactivity was determined in a *γ*-counter (PerkinElmer). Experiments were performed in triplicate. Inhibitory concentration of 50% (IC50) values was determined by GraphPad Prism software (version 5.0).

### 2.6. Cell Uptakes

PC-3 cells were suspended in 500 *μ*L RPMI 1640 and seeded in 24-well tissues culture plates (1 × 10^5^ cells per well) for overnight incubation. After washing with PBS, the cells were incubated with 370 KBq [^68^Ga] Ga-NOTA-MAL-FSH1 in the presence or absence of unlabeled FSH1 (to a final concentration of 1 *μ*M) at 37°C for 15, 30, 60, and 120 min, respectively. The cells were washed 3 times with ice-cold PBS and lysed in 0.5 mL of 1.0 M NaOH. The remaining radioactivity was measured in the *γ*-counter. Cell uptakes were expressed as the percentage of added radioactive dose (% AD). Experiments were performed in triplicate.

### 2.7. MicroPET Imaging

Mice was injected intravenously with 100 *μ*L 3.7 MBq [^68^Ga] Ga-NOTA-MAL-FSH1 in saline under isoflurane anesthesia and subjected to MicroPET imaging. The breathing and body temperature of the mice were monitored by BioVet CT1 system (M2M Imaging Corp.). For the blocking experiment, unlabeled peptide (10 mg/kg body weight) and 3.7 MBq [^68^Ga] Ga-NOTA-MAL-FSH1 were coinjected into five mice. Static PET images were acquired for 5 min using an Inveon MicroPET/CT scanner (Siemens Medical Solutions) at 30 min, 1 h, and 2 h postinjection. The quantification analysis of PET images was performed using the same method as previously reported [13].

### 2.8. Biodistribution Studies

A number of twenty tumor bearing mice were euthanized with isoflurane (inhalation excess) and dissected at 0.5 h, 1 h, and 2 h (5 mice per time-point) after administrating 740 KBq [^68^Ga] Ga-NOTA-MAL-FSH1. To determine the specific uptake, mice were coinjected with the probe with 100 *μ*g of unmodified peptide and sacrificed at 1 h p.i. Normal organs and tumors were collected and weighed. The radioactivity was measured by the *γ*-counter and the percentage of injected dose per gram of tissue (% ID/g) was determined.

### 2.9. Statistical Analysis

Statistical analyses were performed using GraphPad Prism (version 5.0). Data were analyzed using the unpaired, 2-tailed Student's *t*-test. Differences at the 95% confidence level (*p* < 0.05) were considered to be statistically significant.

## 3. Results

### 3.1. Chemistry and Radiochemistry

The labeling was performed within 20 min, with a decay-corrected yield of 93.2 ± 2.1% (*n* = 5) and a radiochemical purity of more than 95%. The specific activity was determined to be at least 25 GBq/*μ*mol.

### 3.2. In Vitro Characterization

The in vitro stability of the ^68^Ga labeled tracer was evaluated by incubating in PBS or human serum at 37°C, respectively. Protein-binding fraction was determined to be 16.5 ± 2.5%. The probe displayed high in vitro stability and showed no degradation products or release of ^68^Ga whether in PBS or human serum (Figures 3(b) and 3(c)). The radiochemical purities of [^68^Ga] Ga-NOTA-MAL-FSH1 were greater than 95% after 2 h in vitro incubation (Table 1).

The partition coefficient (log *D*) of [^68^Ga] Ga-NOTA-MAL-FSH1 was determined to be −3.12 ± 0.05.

### 3.3. Cell-Binding Assay

**Figure 4:**
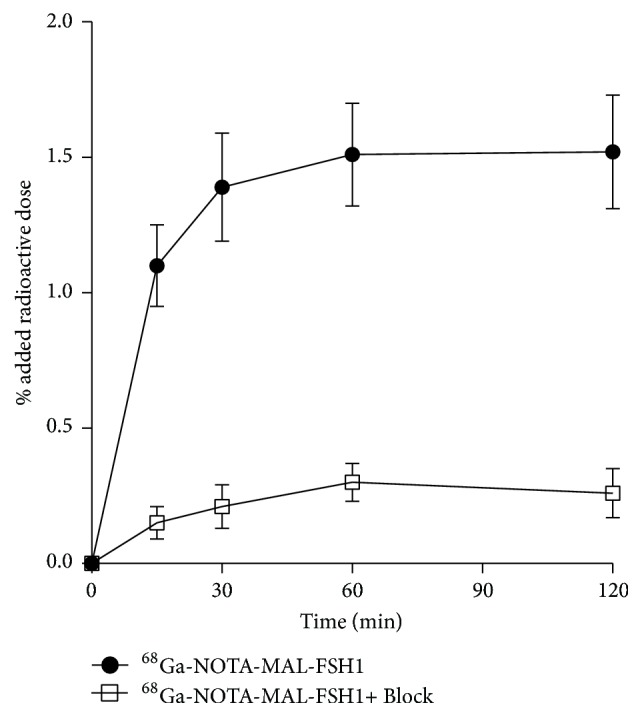
Cell uptake assays of [^68^Ga] Ga-NOTA-MAL-FSH1 in PC-3 cells.

The binding of [^68/nat^Ga] Ga-NOTA-MAL-FSH1 to the FSHR on PC-3 tumors was inhibited by various concentrations of nonlabeled FSH1, and the IC50 values were 123.7 ± 1.21 nM (Figure 4).

### 3.4. Cell Uptake

**Figure 5:**
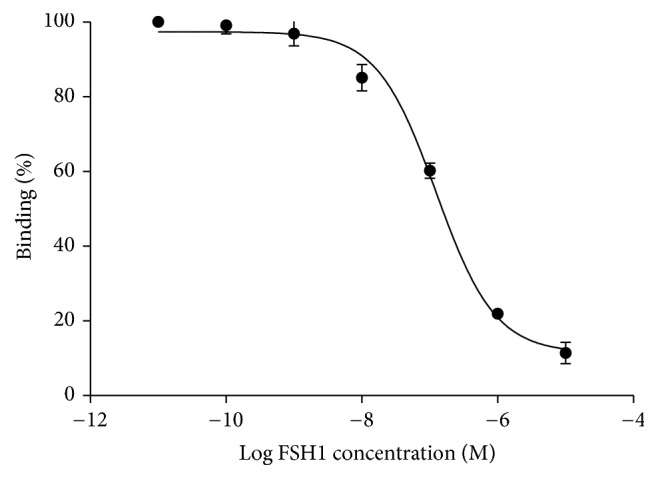
Competition of specific bindings of [^68^Ga] Ga-NOTA-MAL-FSH1 with FSH1.

As shown in Figure 5, [^68^Ga] Ga-NOTA-MAL-FSH1 uptakes in PC-3 cells were 1.10 ± 0.15, 1.39 ± 0.20, 1.51 ± 0.19, and 1.52 ± 0.21% AD for incubating at 15, 30, 60, and 120 min at 37°C, respectively. In the presence of blocking agent, the cell uptakes were significantly reduced to 0.30 ± 0.07% AD after incubating at 60 min, indicating the receptor targeting specificity of the probe.

### 3.5. MicroPET Imaging

**Figure 6:**
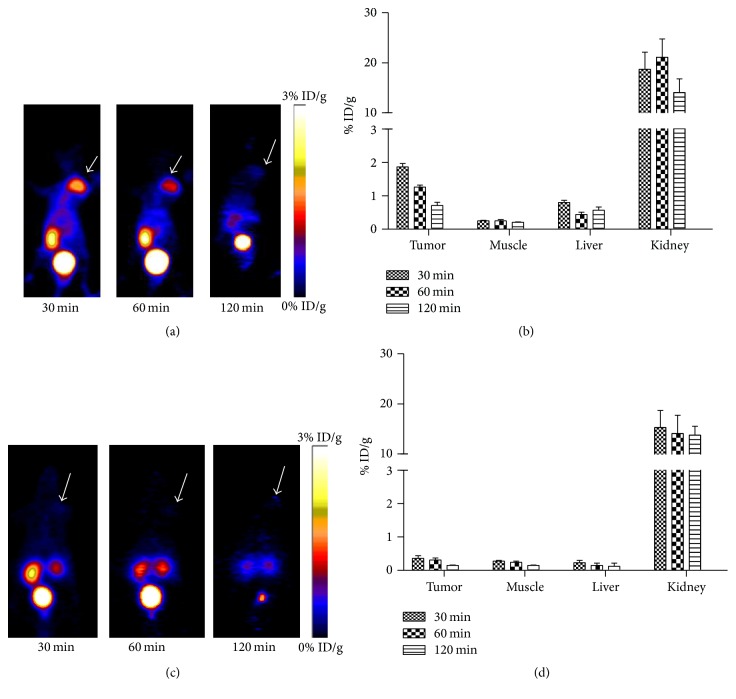
In vivo PET imaging of PC-3 xenografted mice with [^68^Ga] Ga-NOTA-MAL-FSH1. Decay-corrected whole-body coronal MicroPET images of PC-3 tumor bearing mice at 30, 60, and 120 min after injection of 3.7 MBq [^68^Ga] Ga-NOTA-MAL-FSH1 without (a) and with (c) blocking dose. Tumors are indicated by arrows. Quantification of [^68^Ga] Ga-NOTA-MAL-FSH1 in PC-3 tumor, liver, kidneys, and muscle without (b) and with (d) blocking dose. ROIs are shown as mean % ID/g ± SD.

Representative coronal MicroPET images of PC-3 tumor bearing mice (*n* = 5 per group) at different times after intravenous injection of 3.7 MBq [^68^Ga] Ga-NOTA-MAL-FSH1 are shown in Figure 6. PC-3 xenografts were visible with high tumor-to-background contrast. The tumor uptake of [^68^Ga] Ga-NOTA-MAL-FSH1 was determined to be 1.87 ± 0.10, 1.26 ± 0.06, and 0.71 ± 0.10% ID/g at 30, 60, and 120 min (Table 2).

High activity accumulation was also observed in the kidney (21.10 ± 3.64% ID/g at 1 h p.i.) which indicated that ^68^Ga labeled peptide was mainly cleared via the urinary system. The in vivo FSHR binding property of [^68^Ga] Ga-NOTA-MAL-FSH1 was also confirmed by blocking studies. Injection of a large excess of unlabeled FSH1 decreased PC-3 tumor uptakes to 0.31 ± 0.04% ID/g at 1 h postinjection.

### 3.6. Biodistribution Studies

The results of the biodistribution studies are summarized in Table 3. The tumor uptake of [^68^Ga] Ga-NOTA-MAL-FSH1 was significantly higher than those in the blood and normal organs such as heart, liver, spleen, bone, muscle, and testis at 0.5 h, 1 h, and 2 h postinjection (*p* < 0.01). Due to longer retention of the tracer in tumor, the tumor to blood and tumor to muscle uptake ratios were 1.77 ± 0.70, 7.94 ± 1.35, and 10.37 ± 1.16 and 7.42 ± 0.46, 26.13 ± 2.99, and 36.40 ± 2.54, respectively.

Coinjection of excess FSH1 along with [^68^Ga] Ga-NOTA-MAL-FSH1 resulted in significantly reduced radioactivity accumulation in tumor from 1.20 ± 0.27% ID/g to 0.35 ± 0.12% ID/g at 1 h postinjection.

## 4. Discussion

The recent introduction of ^68^Ga PET imaging into clinical practice represents a landmark in the ongoing developments in functional and metabolic imaging that is independent of the availability of a cyclotron [20]. Site-specific labeling is important as it provides chemically uniform radioconjugates with well-defined in vivo properties. Maleimide-NOTA is a NOTA-derivatized chelating system, which has specific reactivity toward thiol group [20]. In this study, NOTA-MAL was conjugated to the cysteine residue of FSH1 for site-specific labeling.

Previous studies have documented the overexpression of FSHR in human prostate cancer tissues, particularly in relation to hormone-resistant prostate tumors [9]. Human prostate cancer PC-3 is a hormone-resistant prostate tumor. Thus the characteristics of [^68^Ga] Ga-NOTA-MAL-FSH1 were preliminary investigated in a PC-3 tumor model.

[^68^Ga] Ga-NOTA-MAL-FSH1 could be achieved in about 20 min through solid-phase extraction with satisfactory radiochemical purity. Our previous studies showed that the radiolabeling yields of ^18^FAl labeled FSH1 and its derivatives, FSH2, were 48.6 ± 2.1% and 41.46 ± 10.36% (nondecay-corrected), respectively, after 30 min preparation. After decay-corrected determination, the labeling yield of ^18^FAl labeled FSH1 and its derivatives was 58.6 ± 2.5% and 50.5 ± 11.2%, respectively. Compared with them, the decay-corrected labeling yield (~90%) of ^68^Ga-NOTA-MAL-FSH1 was significantly higher than those of ^18^FAl labeled counterpart [13, 14].

The radiolabeled NOTA-MAL-FSH1 was hydrophilic as indicated by the negative partition coefficients, which is similar to those of ^18^FAl labeled FSH1 (−3.12 ± 0.05 versus −3.05 ± 0.16, resp.). [^68^Ga] Ga-NOTA-MAL-FSH1 showed in vitro stability in human serum and PBS for at least 2 hours.

The IC50 values of displacement [^68^Ga] Ga-NOTA-MAL-FSH1 with FSHR antagonist, FSH1, were similar to ^18^FAl labeled counterparts, which primarily indicated the affinity of the tracer for FSHR was retained (123 nM versus 252 nM, resp.). In vitro cell uptake assays also showed that coincubation with excess unlabeled FSH1 significantly blocked tumor uptake demonstrating the receptor binding specificity of the PET probe.

After labeling with ^68^Ga, we first performed MicroPET scans for [^68^Ga] Ga-NOTA-MAL-FSH1 in the PC-3 prostate tumor xenograft model. The tracer showed prominent uptakes in the tumor compared with normal tissues such as heart, brain muscle, and livers except kidneys. It was also noted that the uptake values in PC-3 xenografts were slightly lower than those of ^18^FAl labeled FSH1 (1.26 ± 0.06% ID/g versus 2.53 ± 0.20% ID/g at 60 min p.i., resp., *p* < 0.05) [13]. The detailed mechanism was under investigation.

The results of the biodistribution study were consistent with the findings of PET imaging. It showed clear reduction of the radioactivity in the normal organs. The muscle uptakes of the probe were slightly lower than those of ^18^FAl-NOTA-MAL-FSH1 (0.07 ± 0.01 versus 0.15 ± 0.04% ID/g at 1 h p.i., resp.). The resulting tumor to muscle uptake ratio of the probe was more favorable than those of ^18^FAl labeled FSH1 peptides (26.13 ± 2.99 versus 16.17 ± 3.29 at 1 h p.i., resp.) [13]. It indicated that ^68^Ga labeled agent might be an alternative to ^18^FAl labeled compounds for PET imaging of FSHR expression where a cyclotron is of limited to access.

The FSHR specificity of [^68^Ga] Ga-NOTA-MAL-FSH1 was also confirmed by effective tumor uptake inhibition in the presence of excess FSH1 in both noninvasive PET imaging and biodistribution studies. The blocking study suggested that the tumor uptake of the tracer was specific to FSHR.

## 5. Conclusion

In summary, we have successfully developed a radiolabeled tracer, [^68^Ga] Ga-NOTA-MAL-FSH1 with high yield and purity. Preclinical data indicates that [^68^Ga] Ga-NOTA-MAL-FSH1 is promising for noninvasive visualization of FSHR expression in vivo. Further investigation of this novel radiotracer for detection of other FSHR tumor models such as breast and ovarian cancers is currently underway.

## Figures and Tables

**Table 1:** Radiochemical purity (%) of [^68^Ga] Ga-NOTA-MAL-FSH1 after 0.5, 1, and 2 h incubation in PBS and human serum, respectively.

Medium	Time (h)		
	0.5	1	2
PBS	96.7 ± 1.3	97.3 ± 1.2	97.9 ± 1.5
Serum	97.8 ± 0.9	96.9 ± 1.6	96.6 ± 1.1

**Table 2:** Radioactivity accumulation (data obtained from MicroPET) in selected organs of PC-3 tumor bearing nude mice after injection of [^68^Ga] Ga-NOTA-MAL-FSH1 at different time points (*n* = 5/group, mean ± SD).

Uptake (% ID/g)	[^68^Ga] Ga-NOTA-MAL-FSH1			[^68^Ga] Ga-NOTA-MAL-FSH1 block		
	0.5 h	1 h	2 h	0.5 h	1 h	2 h
Tumor	1.87 ± 0.10	1.26 ± 0.06	0.71 ± 0.10	0.36 ± 0.08	0.31 ± 0.04	0.15 ± 0.03
Muscle	0.25 ± 0.02	0.21 ± 0.04	0.13 ± 0.03	0.28 ± 0.09	0.24 ± 0.03	0.11 ± 0.01
Liver	0.80 ± 0.07	0.44 ± 0.06	0.57 ± 0.12	0.23 ± 0.07	0.15 ± 0.03	0.12 ± 0.06
Kidney	18.69 ± 3.44	21.10 ± 3.64	14.05 ± 2.76	15.27 ± 2.89	14.10 ± 3.01	13.78 ± 1.96

**Table 3:** Biodistribution of [^68^Ga] Ga-NOTA-MAL-FSH1 in PC-3 tumor bearing mice at various times after injection (*n* = 5).

Parameter	30 min	60 min	120 min	60 min block
% ID/g in				
Blood	0.95 ± 0.01	0.17 ± 0.02	0.13 ± 0.06	0.19 ± 0.30
Brain	0.07 ± 0.01	0.03 ± 0.01	0.02 ± 0.01	0.03 ± 0.01
Heart	0.35 ± 0.18	0.14 ± 0.05	0.05 ± 0.03	0.14 ± 0.05
Liver	0.93 ± 0.28	0.56 ± 0.04	0.37 ± 0.05	0.12 ± 0.02
Spleen	0.33 ± 0.03	0.26 ± 0.08	0.11 ± 0.05	0.15 ± 0.13
Lung	0.67 ± 0.07	0.14 ± 0.01	0.10 ± 0.01	0.17 ± 0.05
Kidney	24.98 ± 1.77	18.18 ± 2.81	13.91 ± 0.83	15.26 ± 1.20
Stomach	0.40 ± 0.17	0.16 ± 0.06	0.04 ± 0.01	0.18 ± 0.08
Intestine	0.32 ± 0.06	0.15 ± 0.03	0.05 ± 0.02	0.20 ± 0.02
Muscle	0.26 ± 0.01	0.07 ± 0.01	0.04 ± 0.02	0.10 ± 0.02
Pancreas	0.94 ± 0.13	0.11 ± 0.03	0.04 ± 0.02	0.19 ± 0.04
Testis	0.24 ± 0.02	0.08 ± 0.01	0.03 ± 0.01	0.06 ± 0.00
Fat	0.40 ± 0.01	0.13 ± 0.06	0.04 ± 0.00	0.17 ± 0.15
Bone	0.34 ± 0.06	0.15 ± 0.07	0.06 ± 0.02	0.16 ± 0.07
Tumor	1.97 ± 0.17	1.20 ± 0.27	0.97 ± 0.15	0.35 ± 0.12
Ratio of tumor to				
Blood	1.77 ± 0.70	7.94 ± 1.35	10.37 ± 1.16	1.54 ± 0.07
Muscle	7.42 ± 0.46	26.13 ± 2.99	36.40 ± 2.54	1.95 ± 0.23
Liver	2.67 ± 0.50	3.10 ± 0.40	2.84 ± 0.90	3.09 ± 0.24
Intestine	6.68 ± 1.41	11.21 ± 2.42	14.17 ± 1.47	1.74 ± 0.09
Kidney	0.07 ± 0.01	0.10 ± 0.02	0.06 ± 0.00	0.02 ± 0.01
